# Different substitutions at the same β-myosin residue underlie divergent molecular phenotypes and distinct cardiomyopathies

**DOI:** 10.1016/j.jbc.2026.113585

**Published:** 2026-09-23

**Authors:** Sarah J. Lehman, Artur Meller, Shahlo O. Solieva, Jeffrey M. Lotthammer, Lina Greenberg, Stephen J. Langer, Michael J. Greenberg, Jil C. Tardiff, Gregory R. Bowman, Leslie Leinwand

**Affiliations:** 1 Molecular, Cellular, and Developmental Biology, University of Colorado, Boulder, Colorado, USA; 2 Department of Biochemistry and Molecular Biophysics, Washington University in St Louis, St Louis, Missouri, USA; 3 Medical Scientist Training Program, Washington University in St Louis, St Louis, Missouri, USA; 4 Department of Biochemistry and Biophysics, University of Pennsylvania, Philadelphia, Pennsylvania, USA; 5 Department of Biomedical Engineering, University of Arizona, Tucson, Arizona, USA

**Keywords:** myosin, cardiomyopathy, recombinant protein expression, molecular motor, cardiac hypertrophy

## Abstract

In genetic cardiomyopathies, a frequently described phenomenon is how similar mutations in one protein can lead to discrete clinical phenotypes. One example is illustrated by two mutations in beta myosin heavy chain (MYH7) that are linked to hypertrophic cardiomyopathy (HCM) (Ile467Val, I467V) and left ventricular noncompaction (LVNC) (Ile467Thr, I467T). To investigate how these missense mutations lead to independent diseases, we studied the molecular effects of each mutation using recombinant human MYH7 Subfragment 1 (S1) in *in vitro* assays. Both HCM-I467V and LVNC-I467T S1 mutations exhibited similar mechanochemical functions, including unchanged actin-activated maximal ATPase and enhanced actin velocity. Molecular dynamics simulations revealed that both I467T and I467V allosterically disrupt interactions between myosin and ADP, which may contribute to the enhanced actin velocity observed in these mutations. However, I467V and I467T had distinct effects on the single turnover rate of myosin. HCM-I467V S1 showed no change in single ATP turnover rate, while LVNC-I467T reduced the single turnover rate by 50%. These contrasting molecular effects may lead to contractile dysregulation that initiates LVNC-associated signaling pathways that progress the phenotype. Taken together, our results suggest that phenotypic complexity likely originates at the biochemical level.

The most common genetic cardiomyopathy is Hypertrophic Cardiomyopathy (HCM), typically characterized by thickened ventricular walls and impaired relaxation. This disease affects between 1 in 200 to 1 in 500 people in the United States and is the most common cause of sudden cardiac death in people under 30 years old. A leading cause of HCM are mutations in sarcomeric protein genes, and mutations in MYH7 (also known as cardiac myosin heavy chain) account for ∼30% of HCM (1, 2). Until recently, HCM lacked any pharmaceutical intervention that targeted the molecular cause of disease, with existing treatments addressing symptoms of the disease by improving cardiac relaxation (3). However, the FDA approval of two myosin-targeted therapies, Camzyos© (4) and Myqorzo, (5), for the treatment of obstructive HCM highlights the benefit of directly modulating sarcomere function to treat this complex disease.

Another form of cardiomyopathy that can be caused by mutations in MYH7 is left ventricular non-compaction (LVNC) (6, 7, 8). In contrast to HCM, LVNC is a much less understood and studied disease. It is characterized by abnormal growth of cardiac tissue, with excessive deposition but reduced compaction of trabeculae, resulting in a non-compacted, spongiform-like myocardial wall (for review (9)). The introduction of cardiac MRI in the clinic improved the identification of this non-compacted phenotype from thickened, dense muscular wall commonly seen in HCM. While this morphology is characteristic of the disease, the functional phenotype is highly variable. LVNC may present with preserved cardiac function or altered function, including systolic dysfunction, diastolic dysfunction, or a combination of both (10). Thus, clinical management of disease is dependent on the clinical manifestation of the LVNC phenotype. Despite these variable presentations, the natural history of LVNC has been frequently associated with arrhythmias, heart failure, cardiac transplants, and risk of sudden cardiac death. Additionally, due to the spongiform-like myocardium, patients are at risk of thromboembolic events and typically require blood thinners as preventative treatment (11, 12).

Although mutations in the *MYH7* gene have been identified as the cause of disease in many cases, there still is no consensus on how mutations lead to specific cardiomyopathies. Numerous mutations, including those associated with HCM, have been shown to alter the mechanochemical function of myosin, typically enhancing ATPase activity of the motor domain (13, 14, 15). Other mutations increase the number of myosin heads that are available to bind to actin and form force-generating cross-bridges (16, 17, 18). This dysregulation of the relaxed states of myosin may be driven by structural rearrangements of myosin heads (19, 20, 21, 22, 23) and/or biochemical function of myosin (13, 24, 25, 26). Specifically, myosin is known to exist in two distinct states known as the actin-available, disordered (DRX) state and the energy-conserving, super-relaxed (SRX) state of myosin (24, 27, 28). Multiple studies have shown a mutation-specific and disease-dependent shift in the SRX/DRX ratio, providing an additional biophysical mechanism for altered contractility and energetic expenditure in these cardiomyopathies (17, 21, 24). In contrast to HCM, the molecular mechanisms that underlie the complex morphogenetic remodeling and altered cardiac function in LVNC are not well understood. Of note, one study investigated the primary insults caused by LVNC-associated MYH7 M531R, describing an increased power output driven by enhanced mechanochemical activity of the motor (29), despite reduced contractile performance reported in patients expressing this mutation (30).

Here, we investigate how two missense mutations at the same position in MYH7 lead to two different diseases. Specifically, we investigate the biochemical mechanisms that govern how Ile467Val (I467V) causes HCM while Ile467Thr (I467T) causes LVNC. We performed a variety of biochemical assays that probed how these mutations affect intrinsic enzymatic function, actin sliding, and myosin-based relaxation properties. We also connect our experimental results to molecular dynamics (MD) simulations of the β-myosin motor domain carrying the I467T and I467V mutations to understand how a small structural perturbation can alter emergent ensemble properties that give rise to biochemical differences.

## Results

### Actin-activated ATPase activity

The enzymatic function of cardiac muscle contraction is driven by the mechanochemical activity of actomyosin interactions. To assess whether these similar point mutations alter the enzymatic function of the myosin motor, we performed an NADH-coupled actin-activated ATPase assay to measure maximal ATPase activity and actin affinity (Fig. 1). The maximum ATPase activity of the HCM-I467V and LVNC-I467T S1 constructs were unchanged as compared to WT MYH7 S1 (1.6 ± 0.32 s^−1^, 1.6 ± 0.57 s^−1^, and 1.5 ± 0.48 s^−1^, respectively) (Table 1). The actin affinity was unchanged for both the LVNC I467T S1 and HCM-I467V S1 as compared to WT MYH7 S1 (48.2 ± 28.3 μM, 72.7 ± 33.6 μM, and 59.3 ± 31.1 μM, respectively). These data suggest that though these mutations are in close proximity to the ATP-binding pocket, the actin-activated ATPase activity of the myosin motor domain is preserved.

**Figure 1:**
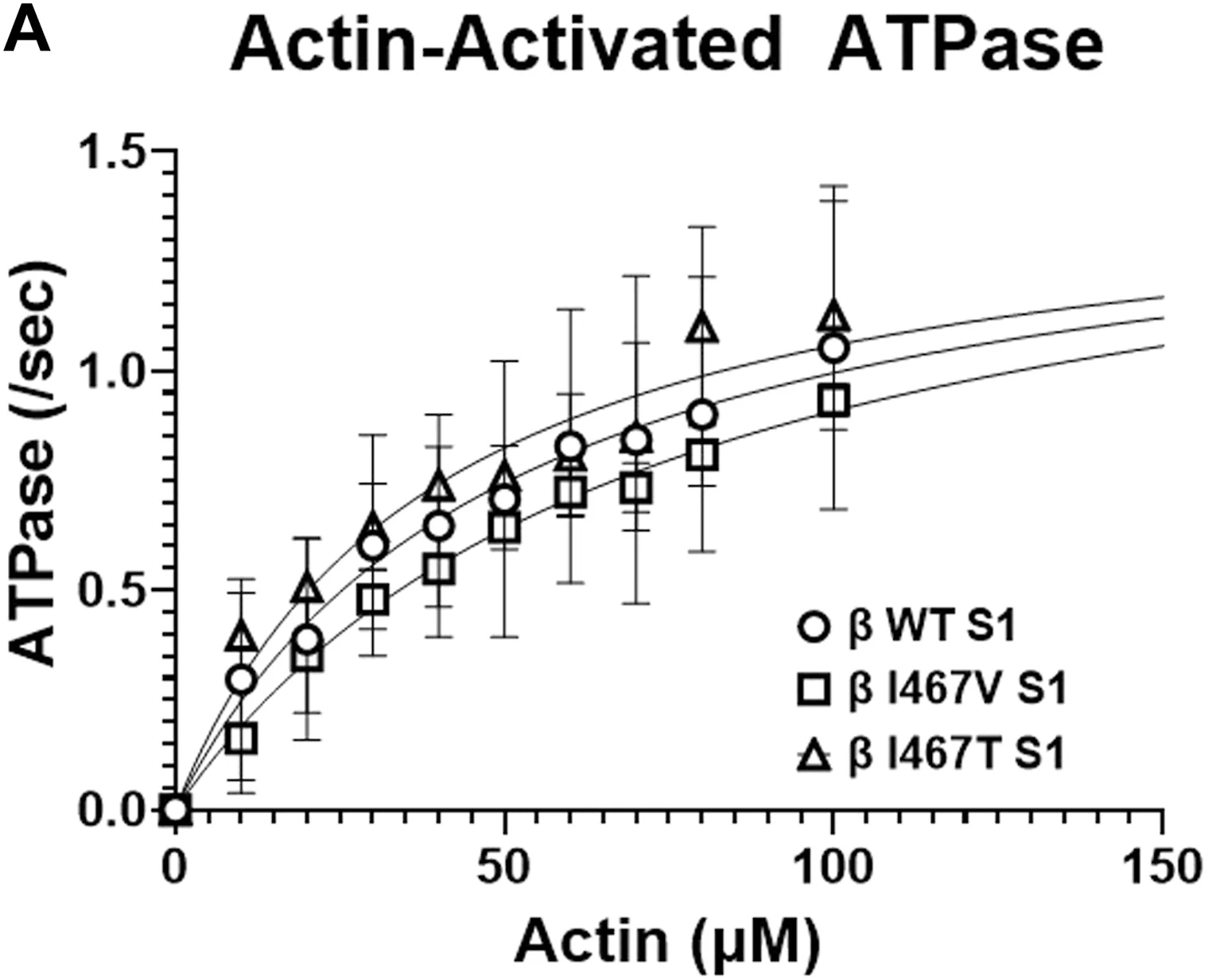
**Actin-activated ATPase Activity of myosin constructs.***A*, ATPase activity of myosin S1 constructs was measured over time with increasing actin concentrations. Data points were fitted using Michaelis-Menten kinetics to obtain maximum ATPase activity (k_cat_) and actin affinity (K_M_). N = 6 to 11 curves from at least 4 individual preparations per myosin S1 construct.

**Table 1:** Summary of actin-activated ATPase activity of myosin S1 constructs

	β WT S1		β I467V S1		Β I467T S1	
	Average	S.D.	Average	S.D.	Average	S.D.
K_cat_ (s^−1^)	1.5	0.48	1.6	0.32	1.6	0.57
K_M_ (μM)	59.3	31.1	72.7	33.6	48.2	28.3

Data are represented as mean ± S.D. N = 6 to 11 curves per myosin S1 construct. A one-way ANOVA was used to assess statistical significance and adjusted with Tukey’s multiple comparisons to assess differences between mutations. No statistically significant differences were identified.

### Single turnover ATPase activity

A growing number of studies support the role of myosin in regulating the relaxation and activation of cardiac muscle. Using a modified single-turnover ATPase assay, we measured the activity of myosin in an actin-independent state to compare HCM-I467V and LVNC-I467T mutational effects on the relaxed state of myosin (Fig. 2). HCM-I467V S1 did not change the single turnover rate of myosin as compared to WT S1 (0.01 ± 0.0005 s^−1^, 0.016 ± 0.006 s^−1^ respectively, *p* = 0.068) (Fig. 2*B*, Table 2). Interestingly, the I467T mutation reduced the single turnover rate more than 2-fold (0.0044 ± 0.0004 s^−1^, *p* = 0.0005) (Fig. 2*B*, Table 2) and increased the area under the curve (AUC, Fig. 2*C*, Table 2), suggesting a greater reduction of energetic expenditure in this mutation. Unlike the actin-activated ATPase activity described above, LVNC-I467T S1 markedly reduced the single turnover rate of myosin compared to HCM-I467V S1, suggesting mutation-specific regulation of myosin independent of actin activation.

**Figure 2:**
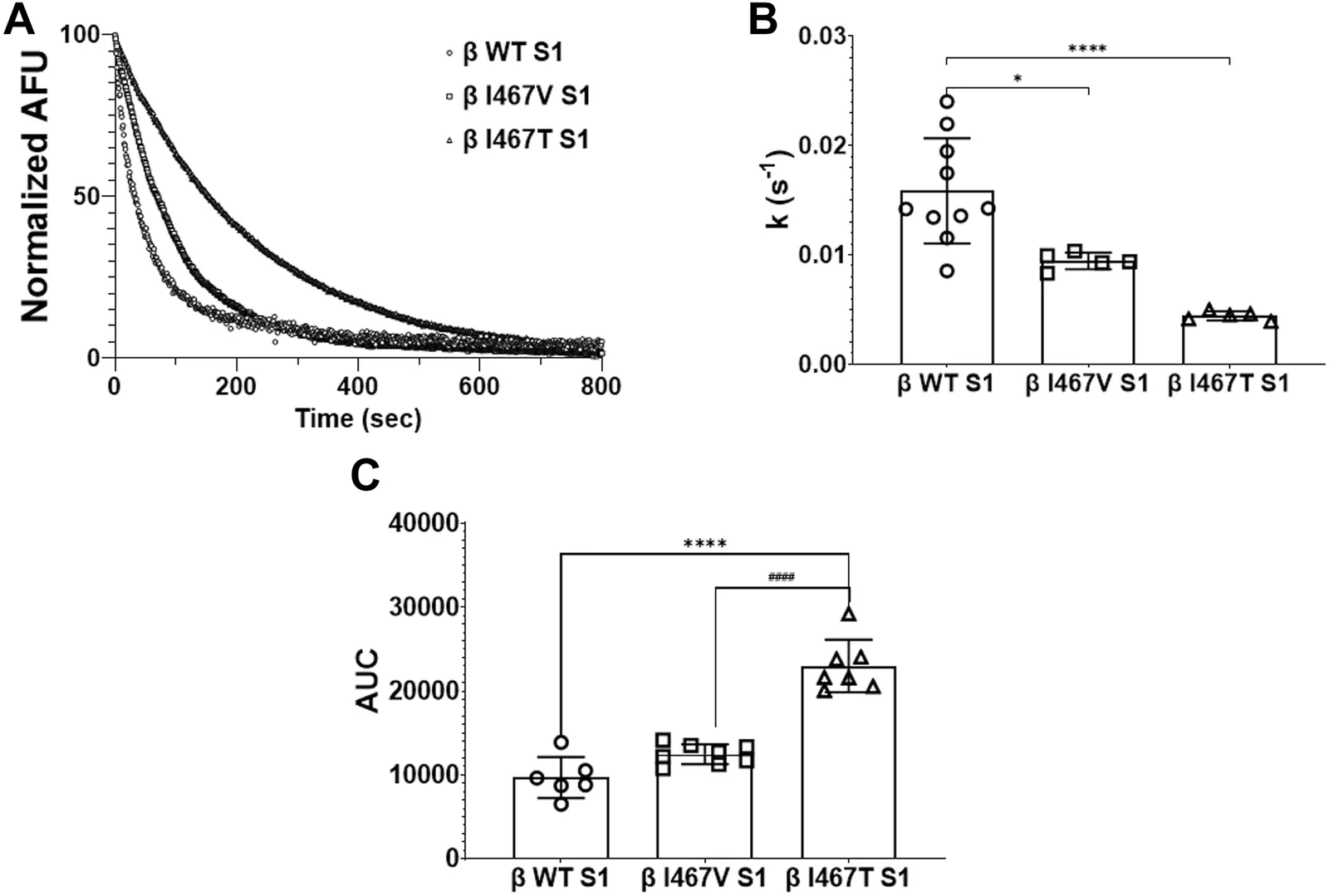
**Single turnover ATPase assay of myosin S1.***A*, representative single-exponential fluorescence decay curves from the single-turnover mant-ATP-dependent ATPase assay for myosin S1 constructs (β-WT: *open circles*, β-I467V: *open squares*, β-I467T: *open triangles*). *B*, the single turnover rate was calculated from the single-exponential fit of decay curves. *C*, area under the curve (AUC) was calculated for each decay curve. A one-way ANOVA was used to assess statistical significance (∗∗∗*p* = 0.0005, ∗∗∗∗ = *p* < 0.0001 *versus* β-WT S1) and adjusted with Tukey’s multiple comparisons to assess differences between mutations (# = *p* < 0.0001 *versus* β-I467V S1). Values are calculated from 5 to 10 decay curves from at least 4 individual preparations per S1 construct.

**Table 2:** Summary table of the single turnover assay of myosin S1 constructs

	β WT S1		β I467V S1		Β I467T S1	
	Average	S.D.	Average	S.D.	Average	S.D.
k (s^−1^)	0.016	0.002	0.010	0.0002	0.0044∗∗∗	0.0002
Lifetime (sec)	72.2	28.2	99.9∗∗∗∗	5.0	226.9∗∗∗∗^#^	19.9
AUC	9615	2696	11,887	1056	21,528∗∗∗∗^#^	1438

Data are represented as mean ± S.D. A one-way ANOVA was used to assess statistical significance (∗*p* = 0.0108, ∗∗*p* = 0.0073. ∗∗∗∗ = *p* < 0.0001 *versus* β-WT S1) and adjusted with Tukey’s multiple comparisons to assess differences between mutations (# = *p* < 0.0001 *versus* β-I467V S1). Values are calculated from 5 to 10 decay curves per S1 construct.

### *In vitro* motility

The velocity at which myosin moves actin can be used to inform on mutational effects on the mechanical function of myosin. To assess the effects of the HCM-I467V and LVNC-I467T mutations, we utilized an unloaded *in vitro* motility assay and measured the velocity of actin movement for each S1 mutation. Both HCM-I467V and LVNC-I467T S1 increased actin velocity >2-fold (HCM- I467V S1: 3.18 ± 0.55 μm/s, *p* = 0.0025 and LVNC-I467T S1: 3.26 ± 0.18 μm/s, *p* < 0.0001) when compared to WT-S1 (WT MYH7 S1: 1.38 ± 0.33 μm/s) (Fig. 3, Table 3). In combination with the NADH-coupled ATPase activity, these data suggest that HCM-I467V and LVNC-I467T do not uniquely alter the actin-dependent function of the myosin motor.

**Figure 3:**
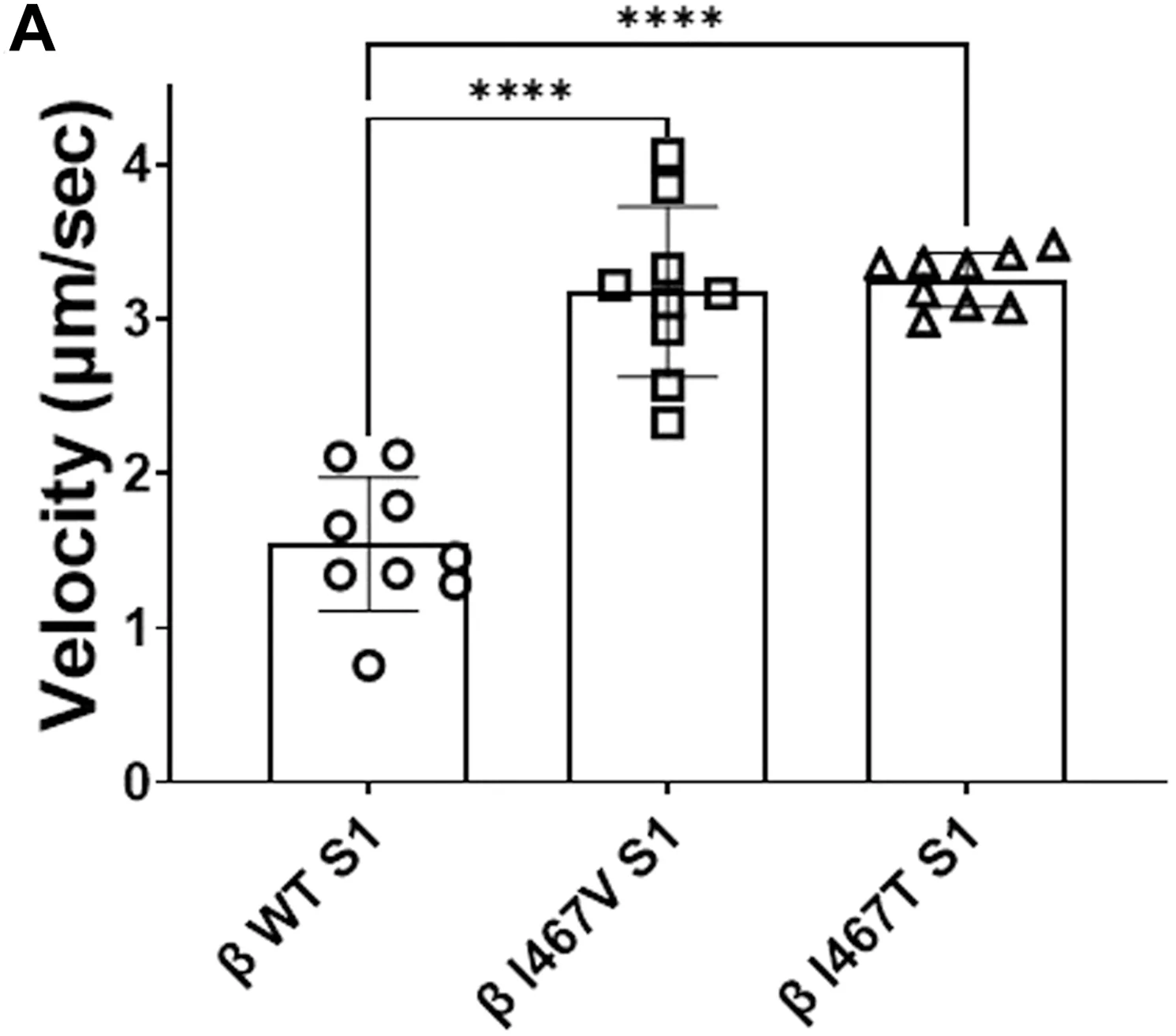
**Unloaded actin sliding velocity.***A*, *In vitro* motility rates for myosin S1 constructs were measured using TRITC-labeled actin filaments. Average actin sliding velocity was calculated from actin filaments that were ≥1 μm in length and mobile in >15 frames per video. A one-way ANOVA was used to assess statistical significance (∗∗∗∗ = *p* < 0.0001) compared to β-WT S1. N = 7 to 8 videos from at least 4 individual preparations per myosin S1 construct.

**Table 3:** Summary table for *in vitro* motility actin sliding velocity for myosin S1 constructs

	β WT S1		β I467V S1		Β I467T S1	
	Average	S.D.	Average	S.D.	Average	S.D.
Velocity (μm/sec)	1.38	0.33	3.18∗∗∗∗	0.55	3.26∗∗∗∗	0.18
% Motile	79.3	7.8	79.5	9.8	77.7	11.8

Values are reported as mean ± S.D. A one-way ANOVA was used to assess statistical significance (∗∗∗∗ = *p* < 0.0001) compared to WT S1. N = 7 to 8 videos per myosin S1 construct.

### Molecular dynamics simulations of I467V and I467T

Both mutants showed no difference in their actin-activated ATPase rates, suggesting that the rate-limiting transition of the cycle (*i.e.*, actin-activated phosphate release) is unchanged by the mutations. At the same time, both mutants showed increased motility speed, and the LVNC-I467T showed a reduction in the single turnover rate, suggesting that the mutations are affecting other key rates of the ATPase cycle. We sought to determine whether these changes could be due to alterations in the structural dynamics of myosin’s active site. The I467T mutation introduces a polar residue at a cluster of hydrophobic residues near the active site that we hypothesized would allosterically weaken interactions between the active site and ADP, thereby accelerating ADP release. The I467V mutation, although a much more conservative mutation, could similarly weaken those interactions by altering residue packing within the hydrophobic cluster. In support of this hypothesis, previous studies have shown that mutations in Switch II can allosterically affect the nucleotide-binding pocket in myosin V (31). Therefore, we sought to test whether a similar trend is observed in β-cardiac myosin that may facilitate faster ADP release.

We used molecular dynamics simulations to investigate the effects of I467T and I467V on β-cardiac myosin dynamics. We ran long simulations of wild-type human β-cardiac myosin, I467T, and I467V starting from a homology model of a pre-powerstroke crystal structure (PDB ID: 5N6A) bound to ADP and Pi. We ran 8 trajectories with an average length of 1300 ns (aggregate time = 10.5 μs) for WT, 47 trajectories with an average length of 1000 ns (aggregate time = 47.7 μs) for I467T, and 10 trajectories each at 1000 ns (aggregate time = 10 μs) for I467V. To understand the effect of the mutation on ADP and Pi contacts, we calculated contact probabilities for ADP to the nucleotide binding pocket and for Pi to neighboring residues. Additionally, we calculated pairwise distances between atoms near the mutation and within the nucleotide-binding pocket.

Although I467 is in Switch II, we found that I467T allosterically disrupts interactions between ADP and the nucleotide-binding pocket. In nucleotide-bound crystal structures of β-cardiac myosin, the nucleotide’s base forms a hydrogen bond with Y134 (Fig. 4*B*). In simulations of WT β-cardiac myosin and the I467V mutant, this hydrogen bond typically remains formed (p_contact_ = 63.9% and 76.1%, respectively, Fig. 4*C*). In contrast, in simulations of I467T, this hydrogen bond is more likely to be broken than formed (p_contact_ = 6.6%, Fig. 4*C*). Furthermore, in nucleotide-bound crystal structures of β-cardiac myosin, there are two residues, W130 and V186, that form a lid above the nucleotide’s base (Fig. 4*B*), likely preventing the nucleotide from leaving the active site. Our simulations suggest that the interaction between W130 and V186 is more likely to break in the I467T and, to a lesser extent, I467V mutants than in the WT ensemble. The contact probability for W130 and V186 is 19.4% in WT but only 0.7% for I467T and 10.6% for I467V (Fig. 4*D*). Simulations of I467T sample partial dissociation events where the ADP is far from its crystal pose (*e.g.*, ADP-N6 to Y134-OH distances of over 1 nm, Fig. 4*E*), but this is not observed in I467V and WT, where the ADP maintains its crystal pose. This is consistent with the greater disruption of the W130-V186 interaction in I467T relative to I467V. Taken together, these results suggest that ADP is not as tightly bound in the nucleotide binding pocket of β-cardiac myosin harboring the I467T mutation and, to a lesser extent, the I467V mutation, providing a structural mechanism for the changes in *in vitro* motility.

**Figure 4:**
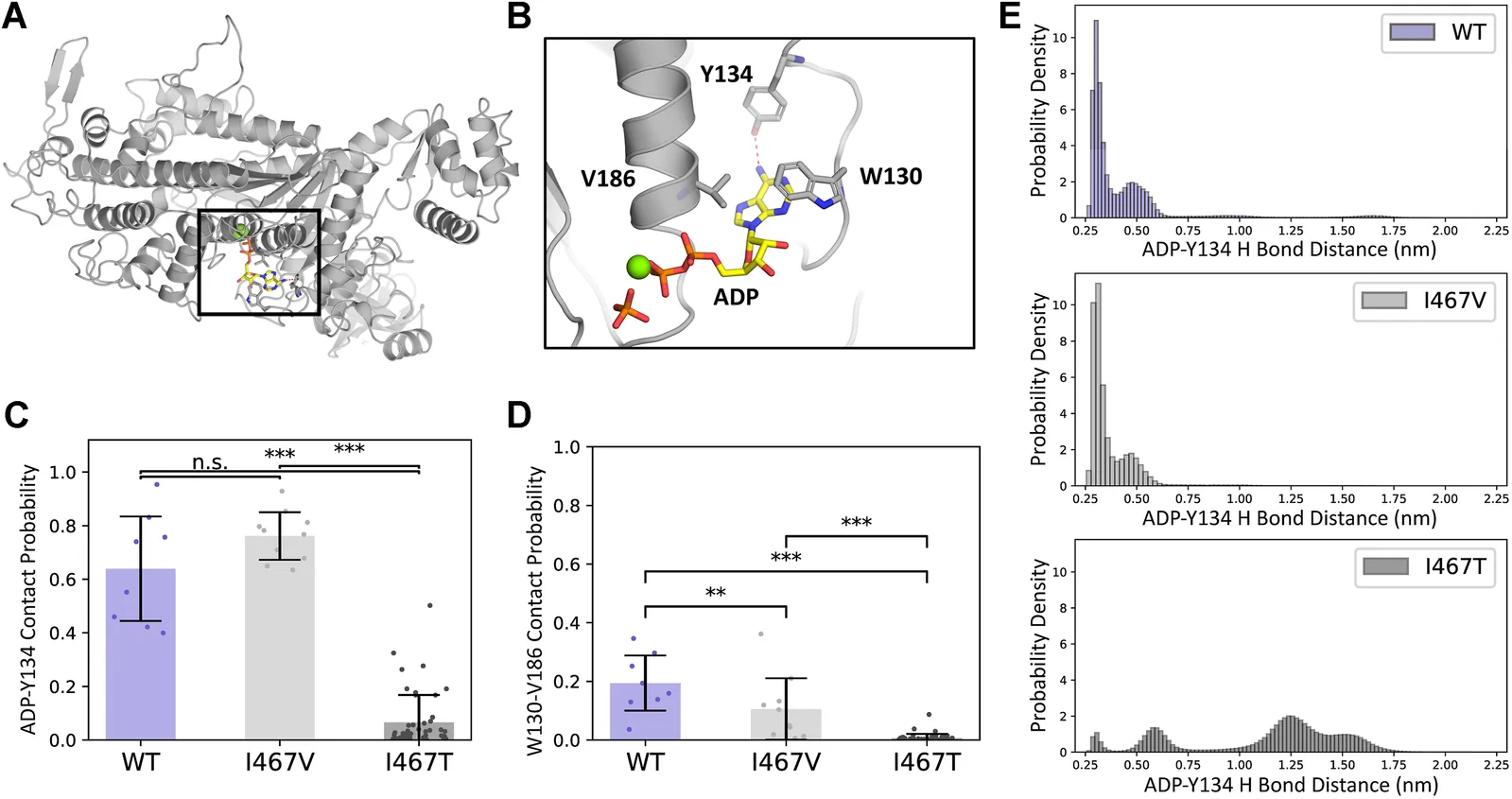
**I467T and I467V disrupt interactions between ADP and the nucleotide-binding pocket in molecular dynamics simulations.***A*, homology model structure of human beta-cardiac myosin, (*B*) focused in on the ADP-Pi binding site, with ADP depicted in *yellow* and *orange*, Pi depicted in *orange*, and Mg^2+^ in *green*. The *red dotted line* indicates a hydrogen bond between adenine and Y134 that is seen in nucleotide-bound crystal structures. *C*, the contact probability between ADP and TYR134 is significantly lower in I467T relative to WT (*p* < 0.001). *D*, the contact probability between W130 and V186, two residues that form a lid over the adenine nucleobase, is significantly lower in I467T (*p* < 0.001) and I467V (*p* < 0.01) relative to WT. *E*, additionally, I467T disrupts a native hydrogen bond between adenine (ADP-N6) and Y134’s hydroxyl group (Y134-OH).

## Discussion

In this work, we investigated two similar missense mutations in β-myosin heavy chain: HCM-linked I467V and LVNC-linked I467T. To investigate how these mutations lead to unique pathologies, we studied the molecular effects of each mutation using recombinantly expressed myosin in various *in vitro* assays. Neither I467V nor I467T altered the maximal actin-activated ATPase activity (Fig. 1), while both mutations showed a >2-fold increase in unloaded actin sliding velocity (Fig. 3). Finally, I467T showed a significant reduction in single turnover rate compared to WT and I467V (Fig. 2). Interestingly, LVNC-I467T slowed the single turnover activity to a rate that is comparable to the ultraslow, SRX state of myosin, suggesting this mutation may impact the biochemical regulation of the relaxation states of myosin.

While hundreds of sarcomeric mutations have been identified and linked to HCM (32, 33), the molecular mechanism by which these mutations converge on disease has not been identified. Historically, HCM was described as a gain-of-function disease as many mutations in MYH7 lead to enhanced mechanochemical function of myosin. While HCM-I467V showed no change in the actin-activated ATPase activity, it did significantly increase the velocity myosin moves actin filaments. It is important to note that these assays are dependent on distinct steps of the mechanochemical cycle. The ATPase assay is an attachment-limited assay in which enzymatic function is limited by phosphate release, while the actin sliding assay is detachment-limited, rate-limited by the release of ADP (34). Though this mutation is located in the phosphate-leaving tunnel of the myosin motor (35, 36), the unchanged k_cat_ reported for this mutation suggests that phosphate release is unchanged in this mutation, whereas the enhanced actin velocity in the HCM-I467V S1 mutation may be due to an increase in ADP release rate. Though we did not observe complete ADP dissociation in molecular dynamic simulations, we found that motors carrying the I467V mutation were more likely to adopt conformations primed for ADP release (*i.e.*, structures with an open “lid”). This result suggests that although the isoleucine does not directly interact with ADP, it allosterically stabilizes interactions between ADP and the nucleotide-binding pocket. Future studies using stopped-flow kinetics and structural techniques are necessary to directly test these computational predictions.

Importantly, the enhanced velocity was measured in the absence of load. Increased velocity in isolation would contribute to increased power and hyperdynamic function in HCM. Additionally, one of the hallmarks of the disease is an inability to relax properly (37). It may be that I467V affects the population of a biochemical DRX-like state, a common finding of HCM-associated myosin mutations (17, 24, 25), contributing to diastolic dysfunction seen in the disease. In support of this, molecular dynamic simulations by Li *et al.* defined a link between the size of the phosphate tunnel (in which this mutation resides) and regulation of the SRX state (38), providing further evidence for primary structure-function disruption for the mutations studied in this work. Future studies, including X-ray diffraction that measure the structural state of myosin under load, will be necessary to 1) define the population of myosin in an auto-inhibited state and 2) determine if these changes contribute to the diastolic dysfunction and hypercontractility described in HCM.

LVNC-I467T S1 exhibited similar mechanochemical function to the HCM-I467V S1 mutation, including unchanged total actin-activated ATPase cycle time (the inverse of k_cat_) and enhanced actin velocity (Figs. 1*A* and 3*A*). Computational modeling by Chakraborti *et al.* suggested that I467T does not alter the rate of ATP hydrolysis of the myosin motor domain (35). While we did not specifically investigate the rate of hydrolysis, our steady-state ATPase measurements demonstrate that if there are changes in the rate of hydrolysis, they must be small enough that they do not change the rate-limiting step of the ATPase cycle. Previous studies have shown that sequence variation in myosin motors allows for regulation of specific functional roles by modulating the probability of structural conformations that are associated with these functions (39, 40). Similar to the I467V mutation, we found that I467T allosterically influenced the opening of the “lid” above the nucleotide-binding pocket of the motor domain, bringing the probability of the formation of that lid down to under 1% and priming for increased ADP release at a level higher than observed in I467V.

The I467T S1 slowed the single turnover activity to a rate that is comparable to the ultra-slow, energy-sparing ATP turnover of the SRX state of β-myosin (24). Chakraborti *et al.* found that I467T stabilized the post-rigor state of myosin, preventing the structural rearrangements necessary for the recovery stroke and pre-power stroke structure that is thought to be required for increasing the ultra-slow SRX state of myosin (23). However, multiple studies have suggested the likely existence of additional structural states that result in ultraslow ATPase activity, but that have not yet been identified (41, 42, 43). Thus, one can imagine that by slowing the recovery stroke of myosin, I467T slows myosin’s progression through the ATPase cycle to a rate comparable to the ultra-slow state of myosin that may or may not be equivalent to the canonical SRX state. Of note, the family affected by this mutation presents clinically with reduced systolic function. Given that velocity of contraction is inversely proportional to force production, the increased unloaded velocity reported for this mutation suggests a reduction in myosin duty ratio and force production. Coupled to the ultraslow single turnover rate measured, we hypothesize that LVNC-linked I467T initiates disease, in part, by reducing sarcomere force production and/or the availability of myosin heads for force-generation. Interestingly, the simulations of I467T show a disruption of interactions between ADP and the nucleotide-binding pocket, which may provide a structural explanation for why basal ATPase activity is slower in I467T and not in I467V. This complex combination of altered mechanochemical function and energetics may trigger a downstream signaling pathway distinct from both HCM and dilated cardiomyopathy, leading to the development of LVNC. However, additional studies using sarcomeric systems under load that allow for autoinhibition of myosin both structurally and functionally are necessary to confirm this proposed mechanism.

Disease pathogenesis in patients is a complex and protean process involving the activation of many adaptive and maladaptive pathways in response to mutation. Moreover, the sarcomere is a highly complex macromolecular complex, and mutations can affect multiple biophysical processes beyond the endpoints examined here. While further studies are required to completely define the mechanisms by which these mutations lead to divergent phenotypes in patients, our work suggests that phenotypic complexity, in part, originates at the biochemical level. Thus, a molecular understanding is likely critical to developing targeted therapies to treat discrete diseases that arise from similar point mutations. By directly modulating the molecular progression of disease, myosin- and/or sarcomere-targeted small-molecule therapies offer a path to treat many patients.

## Experimental procedures

### Recombinant myosin construct generation and protein purification

Human recombinant β myosin S1 constructs (residues 1–842) were used to assess the functional consequences of mutations located within the motor domain. Site-directed mutagenesis was performed using the Agilent Quikchange II XL kit (Agilent Technologies, Santa Clara, CA) was used to individually introduce the I467V and I467T mutations into the S1 motor domain and was confirmed *via* cDNA sequencing. Constructs were tagged with a C-terminal affinity tag complementary to the PDZ binding domain (44). Viral construct, protein generation, and protein purification were performed as described by Lee *et al.* (45). Of note, the human myosin S1 constructs were decorated with mouse essential and regulatory light chains that are expressed in the C2C12 cell line. At least four independent myosin S1 preparations were produced for each myosin construct to be used across the *in vitro* experiments. Tissue purified rabbit skeletal actin was also purified as previously described (45).

### NADH-coupled ATPase assay

Steady-state actin-activated ATPase rates were measured using purified MYH7 S1 fragments expressing either the I467V or I467T mutations using an NADH-coupled regenerative ATPase assay as previously described (45). Purified wild-type (WT) MYH7 S1 was used as a control in all experiments. MYH7 S1 (f.c. = 0.4 μM) was mixed with filamentous actin (concentration range: 0–100 μM) in the following solution: 20 mM HEPES pH 7.0, 25 mM KCl, 5 mM MgCl_2_, 2 mM ATP, 5 mM DTT, 3 mM phosphoenol pyruvate, 1 mM NADH, 0.8 mM pyruvate kinase/lactate dehydrogenase. Absorbance of NADH was monitored at 340 nm over 60 min as a surrogate for ATP hydrolysis and linear absorbance ranges were determined for each condition. Using a NADH-standard curve to calculate the nanomoles of NADH/absorbance and myosin concentration per well, ATPase rates of individual wells were converted from ATP·s^−1^ to per-second rates. Basal ATPase activity (0 μM actin) was subtracted from ATPase rates of all actin concentrations, and the data were fitted with Michaelis-Menten kinetics using GraphPad Prism 9 software. The data were reported as maximum actin-activated ATPase activity (k_cat_, s^−1^) and actin affinity (K_m_, μM).

### Single turnover ATPase assay

To assess the single-turnover ATPase rate, purified recombinant S1 proteins were run in a modified ATPase assay. To deplete ATP from the myosin S1 proteins, proteins were individually dialyzed exhaustively against a buffer containing: 10 mM Tris pH 7.4, 30 mM Potassium Acetate, 1 mM EDTA, and 4 mM MgCl_2_. Myosin S1 (f.c. = 0.4 μM) was rapidly mixed with 0.4 μM mant-ATP (2′-(or-3′)-*O*-(*N*-Methylanthraniloyl) Adenosine 5′-Triphosphate), and the reaction aged for 60 s to allow for mant-ATP + S1 binding and ATP hydrolysis. The reaction was then rapidly mixed with 4 mM Na-ATP, and decay of the mant-ATP signal was immediately monitored using a BMG ClarioSTAR plate reader (BMG Labtech). Specifically, mant-ATP was excited at 385 nm, and emission was monitored at 450 nm at 1 Hz for 1000 s. The normalized decay curves were fitted with a single-exponential decay (constrained fit of Y_0_ = 100 and plateau = 0) using GraphPad Prism 9.0. The ATPase rate (K, s^−1^) and lifetime were calculated for MYH7 I467T and I467V and compared to MYH7 WT. The area under the curve was also calculated for all constructs to assess ATP consumption.

### Molecular dynamics simulations

A homology model of beta-cardiac myosin in the pre-powerstroke state from Meller *et al.* was used as the starting structure for simulations of WT, I467T and I467V.

All simulations were performed in the GROMACS simulation software with the CHARMM36m force field (46, 47). Each simulation was prepared by solvating the myosin motor domain with TIP3P water in a dodecahedron box extending 1 nm around the protein. The system was then neutralized and raised to a salt concentration of 0.1 M NaCl. Energy minimization was performed using steepest descent till the maximum force on any atom was below 1000 kJ/(mol x nm). Equilibration was performed using the Bussi-Parrinello thermostat and the Parrinello-Rahman barostat with all heavy atoms restrained at 300 K.

Production runs were performed at 310 K with the leapfrog integrator, Bussi-Parrinello thermostat, and Parrinello-Rahman barostat. A 12 Å cutoff distance was utilized with a force-based switching function starting at 10 Å. We employed periodic boundary conditions to mimic the effects of bulk solutions. The PME method with a grid density greater than 1.2 Å^3^ was used to calculate long-range electrostatics. All hydrogen bonds were constrained with the LINCS algorithm to enable the use of a constant integration timestep of 2 fs. All simulations were performed on our in-house compute cluster consisting of NVIDIA A5000 nodes.

### Simulation analysis

We used MDTraj’s contacts function, which takes a subset of residues and reports any contacts between their atoms if any two atoms are within a cutoff threshold distance of each other; we defined the cutoff distance as 4 Å and used heavy atoms only. For each trajectory, we calculated the amount of time a contact exists between two residues and divided that by the length of the trajectory, yielding a probability of contact between those residues. We also used MDTraj’s distance function, which calculates distances between any given atoms. Statistical significance was determined using one-way ANOVA between WT and the two mutants followed by *post hoc* Tukey tests. The scripts for calculating contacts, statistical testing, and graphing the data can be found here: https://github.com/ssolieva/i467t.

### *In vitro* motility

*In vitro* motility was used to assess the effect of I467T and I467V mutations on myosin’s ability to translocate actin. Purified recombinant S1 mutants and WT proteins were mixed with filamentous actin in a 1:3 ratio and 1 mM ATP, then subsequently spun at 90,000 rpm for 25 min at 4 °C to remove inactive myosin molecules. The active myosin S1 molecules were diluted to a final concentration of 0.35 μM in a 1X buffer containing: 20 mM HEPES pH 7.5, 25 mM potassium chloride, 5 mM MgCl_2_, 1 mM EGTA, and 10 mM DTT. Glass coverslips were dipped in 0.2% nitrocellulose and adhered to a glass microscope slide to create a flow cell for the experiment. To begin, 3 μM SNAP-PDZ was flowed into the cell and incubated for 2 min at room temperature. 1 mg/ml BSA was then added and incubated for 1 min at room temperature to block all non-specific binding sites. 0.35 μM myosin S1 solution was added to the flow chamber and incubated for 3 min to ensure proper binding to the surface mobilized SNAP-PDZ domain. Excess myosin was washed out, and phalloidin-labeled filamentous actin (f.c. 2–5 nM) was added and incubated for 1 min. Finally, activating solution containing 20 mM HEPES pH 7.5, 25 mM potassium chloride, 5 mM MgCl_2_, 1 mM EGTA, and 10 mM DTT, 15 mM ATP, 4 mg/ml glucose, 0.135 mg/ml glucose oxidase, 0.0215 mg/ml catalase, and 0.5% methylcellulose was added to the flow chamber and incubated for 30 s at room temperature. Using an inverted wide-field microscope (Nikon Inc), movement of the phalloidin-labeled actin was monitored over time. Videos were collected between one and three frames per second and a total of 30 frames were collected for each movie. Using MATLAB, actin filament velocity was calculated for individual filaments and averaged across videos. At least 50 filaments were measured for each mutation, across three separately purified samples for MYH7 WT, I467T, and I467V S1.

### Statistical analysis

For actin-activated ATPase assays, single-turnover ATPase assays, *in vitro* motility assays, a one-way ANOVA was used to assess significant differences. Tukey’s multiple comparisons test was applied *post hoc* to determine the mutation-specific effects in these assays. For simulation analysis, we conducted a Student’s *t* test when the variances were approximately equal and Welch’s *t* test when the variances were unequal. For all comparisons, significance was assigned at *p* > 0.05.

## Data availability

∗Data will be shared upon request. Please contact Leslie Leinwand (Leslie.leinwand@colorado.edu).

## Supporting information

This article contains supporting information.

## Conflicts of interest

The authors declare the following financial interests/personal relationships which may be considered as potential competing interests:

S. J. L. is an employee of Edgewise Therapeutics and holds stock or stock options. L. A. L. is a co-founder and stockholder of Kardigan, Inc.
