# Supplementary material for: Different substitutions at the same β-myosin residue underlie divergent molecular phenotypes and distinct cardiomyopathies
Source: J Biol Chem. 2026 Sep 23;302(11):113585. doi: 10.1016/j.jbc.2026.113585 (PMC13696803; doi:10.1016/j.jbc.2026.113585)
Supplement: Supplementary file 1 [file mmc1.docx]

**Tables**

**Supplemental Table 1. Summary of basal activity of myosin S1 constructs in NADH ATPase assay**.

|  | **β WT S1** | | **β I467V S1** | | **Β I467T S1** | |
| --- | --- | --- | --- | --- | --- | --- |
|  | Average | S.D. | Average | S.D. | Average | S.D. |
| **Basal ATPase (s^-1^)** | 0.12 | 0.08 | 0.24 | 0.1 | 0.20 | 0.13 |

Data are represented as mean ± S.D. N=6-11 curves per myosin S1 construct. A one-way ANOVA was used to assess statistical significance and adjusted with Tukey’s multiple comparisons to assess differences between mutations. No statistically significant differences were identified.
